# P2X7R antagonists in chronic stress-based depression models: a review

**DOI:** 10.1007/s00406-021-01306-3

**Published:** 2021-07-19

**Authors:** Iven-Alex von Muecke-Heim, Clemens Ries, Lidia Urbina, Jan M. Deussing

**Affiliations:** 1 Max Planck Institute of Psychiatry, Molecular Neurogenetics, Munich, Germany; 2 International Max Planck Research School for Translational Psychiatry (IMPRS-TP), Munich, Germany; 3 Graduate School of Systemic Neurosciences, University of Munich (LMU), Munich, Germany

**Keywords:** Animal models, Chronic stress, Microglia and macrophages, P2X7 receptor (P2X7R), P2X7R antagonists

## Abstract

Depression affects around 320 million people worldwide. Growing evidence proposes the immune system to be the core interface between psychosocial stress and the neurobiological and behavioural features of depression. Many studies have identified purinergic signalling via the P2X7 receptor (P2X7R) to be of great importance in depression genesis yet only a few have evaluated P2X7R antagonists in chronic stress-based depression models. This review summarizes their findings and analyses their methodology. The four available studies used three to nine weeks of unpredictable, chronic mild stress or unpredictable, chronic stress in male mice or rats. Stress paradigm composition varied moderately, with stimuli being primarily psychophysical rather than psychosocial. Behavioural testing was performed during or after the last week of stress application and resulted in depressive-like behaviours, immune changes (NLRP3 assembly, interleukin-1β level increase, microglia activation) and neuroplasticity impairment. During the second half of each stress paradigm, a P2X7R antagonist (Brilliant Blue G, A-438079, A-804598) was applied. Studies differed with regard to antagonist dosage and application timing. Nonetheless, all treatments attenuated the stress-induced neurobiological changes and depressive-like behaviours. The evidence at hand underpins the importance of P2X7R signalling in chronic stress and depression. However, improvements in study planning and reporting are necessary to minimize experimental bias and increase data purview. To achieve this, we propose adherence to the Research Domain Criteria and the STRANGE framework.

## Introduction

Depression is characterised by three main symptoms: low mood, anhedonia, and decreased energy [1]. Approximately 320 million or 4.4% of the global population are affected [2]. Women have a twofold increased disease risk compared to men [3]. Depression sits among the top three causes of years lived with disability worldwide [4] and burdens individual life prospects and health care systems alike [5–7]. Treatment employed depends on depression severity and patient response, yet overall yields heterogeneous outcomes [8, 9]. In fact, only 50–70% of depressed patients recover within 1 year of diagnosis [6, 10] and an alarming 15–20% experience a chronic course of depression (> 24 months) [6, 10, 11]. The latter is associated with reduced quality of life, limitations of daily activities, treatment resistance, suicide attempts and comorbidities [12]. Aside the serendipitous discovery of ketamine, no other major therapeutic breakthroughs have been achieved in the past decades [13]. Moreover, no reliable and clinically applicable diagnosis or outcome prediction tools are available [8, 14]. This dire situation urges us to evaluate and optimize preclinical and clinical methods to advance novel therapeutic strategies [9].

Within recent years, the concept of depression has changed and now more than ever includes immunological disease features [15–18]. However, on a mere environmental level, psychosocial stress is still the most common and major risk factor of depression [14, 19–25]. Concerning the neurobiological link between stress and depression, several studies have highlighted the role of microglia and macrophage (M/Ms) activation [18, 26–32] mediated by purinergic signalling via the membrane-bound adenosine triphosphate (ATP) receptor P2X7 (P2X7R) [33–40]. On a genetic level, studies in humans and mice have found an association between polymorphisms in the *P2RX7* gene and depressive symptoms [41–46]. Meanwhile, a growing body of evidence emphasizes the potential of P2X7R as a novel drug target in depression [33, 35, 47–51] and small, placebo-controlled phase II studies investigating P2X7R antagonists in major depressive disorder are ongoing [52, 53]. Yet, only a handful of studies have evaluated P2X7R antagonistic drugs in chronic stress-based depression models [34].

Based on the emerging role of P2X7R as an interface between stress and the immunological features of depression, this review aims to analyse the available studies on pharmacological P2X7R antagonism in translational murine depression models. Although no single approach can represent the plethora of environmental and biological factors of depression, chronic stress-based models are currently considered the most valid option in reflecting disease complexity overall. They reliably induce core depressive symptoms, behaviours and biological changes by exposing animals to unpredictable, mild to moderate environmental stress over several days to weeks. Because of that, they are common models in translational neuropsychiatry [13, 54–56]. We thus included studies using chronic psychosocial and psychophysical stress (CPSS/CPPS) in the form of chronic unpredictable stress (CUS), unpredictable, chronic mild stress (UCMS/CMS) or chronic social defeat (CSD) in this review. After summarizing the evidence revolving around chronic stress, the immune system and P2X7R signalling in depression, we examine the available studies from which we can draw meaningful insights for future research.

## From stress to depression: the emerging role of the immune system

The pathogenesis of depression is complex and has been extensively investigated. Nowadays, explanatory approaches integrate the entirety of endocrine, neurochemical, and plasticity aberrations in a fine-grained framework of environmental influences and epi-/genetic as well as psychosocial vulnerabilities [14, 34, 57]. Since the concept of stress was introduced to the scientific community in 1936 by Hans Selye [58, 59], a strong link between CPSS/CPPS and depression has been reported. Many studies and meta-analyses have demonstrated an elevated depression risk following childhood maltreatment and adverse life events in adolescence and adulthood [21, 60–66]. The biological mechanisms that translate this form of environmental stress into the nosological entity called depression are not yet fully understood, but the immune system, in particular cytokines and microglia, is proposed to be a principal component [16, 35, 67–69]. This notion stems from a larger number of human and rodent studies, which found elevated levels of inflammatory markers including but not limited to C-reactive protein (CRP), interleukin-1-family cytokines like interleukin-1 beta (IL-1β) and IL-18, tumour necrosis factor alpha (TNF-α) and IL-6 in CPSS/CPPS or depression [15, 70–78].

Interestingly and in line with this so-called cytokine hypothesis of depression [16], immunological diseases like asthma or diabetes are associated with elevated inflammatory cytokine levels and an up to twofold increased depression risk [68, 79–82]. On a genetic level, a recent correlation study based on a large genome-wide association study reported an overlap between CRP levels and depression symptoms as well as an association of upregulated IL-6 with suicidality [72]. In 2014, a clinical study found elevated NLR family pyrin domain containing 3 (NLRP3) and caspase-1 gene expression in the blood of *n* = 40 treatment-naive depressed patients. The NLRP3 and caspase-1 expression along with IL-1β and IL-18 levels were ameliorated by amitriptyline treatment [83]. NLRP3 inflammasome assembly, caspase-1 activation and IL-1β release are generally known to be triggered by P2X7R activation, which is one of the main NLRP3 activators [36] and a potent inductor of M/Ms activation and proliferation [84, 85]. Accordingly, animal studies investigating acute and chronic stress in the context of depressive-like states reported altered recruitment and increased activation of M/Ms, predominantly in frontolimbic regions [27, 86–95]. In humans, positron emission tomography studies in depressed patients detected elevated microglia activity in the prefrontal cortex (PFC), anterior cingulate cortex and insula by using a radiotracer for translocator protein [96–98], a transmembrane protein located in the outer mitochondrial membrane that serves as a sensitive neuroinflammation marker [99]. Overall, these findings suit the recent notion of depression as a microgliopathy [26]. It is even discussed whether M/Ms are the key facilitators of the relation between CPSS/CPPS and depression [15, 18, 100]. In line with this, several studies have demonstrated the joint role of IL-1β, IL-6 and TNF-α along with M/Ms activation in tryptophan-kynurenine-pathway disruption, glutamate excitotoxicity, blood brain barrier disruption and neuronal loss in depressive and suicidal behaviour [101–104]. Based on these findings, the P2X7R-NLRP3-IL-1β cascade is proposed to be the primary interface between CPSS/CPPS, humoral and cellular immunity, and depression [15–17, 33–35, 38, 67, 94, 100, 105].

## Neuroinflammation, P2X7R-signalling and chronic stress

Purinergic signalling is a phylogenetically ancient, ubiquitous cellular mechanism involved in cell-to-cell crosstalk, tissue homeostasis and immune functioning [36, 106]. Nearly all cells release purines and bind them with a variety of membrane-bound receptors. The purine receptor family consists of P1 receptors for adenosine and P2 receptors for nucleotides. The latter are again divided into eight metabotropic P2Y and seven ligand-gated ionotropic P2X receptors [36, 107]. The contribution of P2X7R signalling in sterile inflammation and infection has been examined in systemic and brain-specific entities like Parkinson’s or Alzheimer’s disease, bipolar disorder, and schizophrenia [36, 38, 43, 108, 109]. At the pathogenetic intersection of chronic stress and depression, P2X7R signalling has been identified as a pertinent factor in causing the numerous and complex neurobiological aberrations [33, 34, 39, 43, 48].

In the brain, P2X7R is strongly expressed on M/Ms [30, 110, 111] and mainly active in chronic, inflammatory conditions due to its high activation threshold and slow desensitization (half maximal effective ATP concentration: 2–4 micromolar) compared to other P2X receptors [36, 109, 111, 112]. The functional P2X7R is comprised of three congregating P2X7R monomers [36]. In the context of neuropsychiatric research, many studies have found that CPSS/CPPS intensifies glutamate release from neurons, which in return triggers more ATP release from neurons, astrocytes, and microglia [33, 35, 51, 67, 94, 113–116]. In the extracellular space, ATP is a part of damage-associated molecular patterns, which act as molecular distress signals [117, 118]. Following ATP binding, P2X7R allows immediate potassium efflux, sodium and calcium influx and the passage of several large organic cations via an intrinsic macropore function [36, 47, 117]. The long-standing hypothesis of P2X7R pore dilation was recently disproved [36, 117, 119, 120]. Upon sustained P2X7R activation, for example by CPSS/CPPS, apoptosis or necrosis is triggered by caspase-3 cleavage [34, 47, 117].

**Fig. 1:**
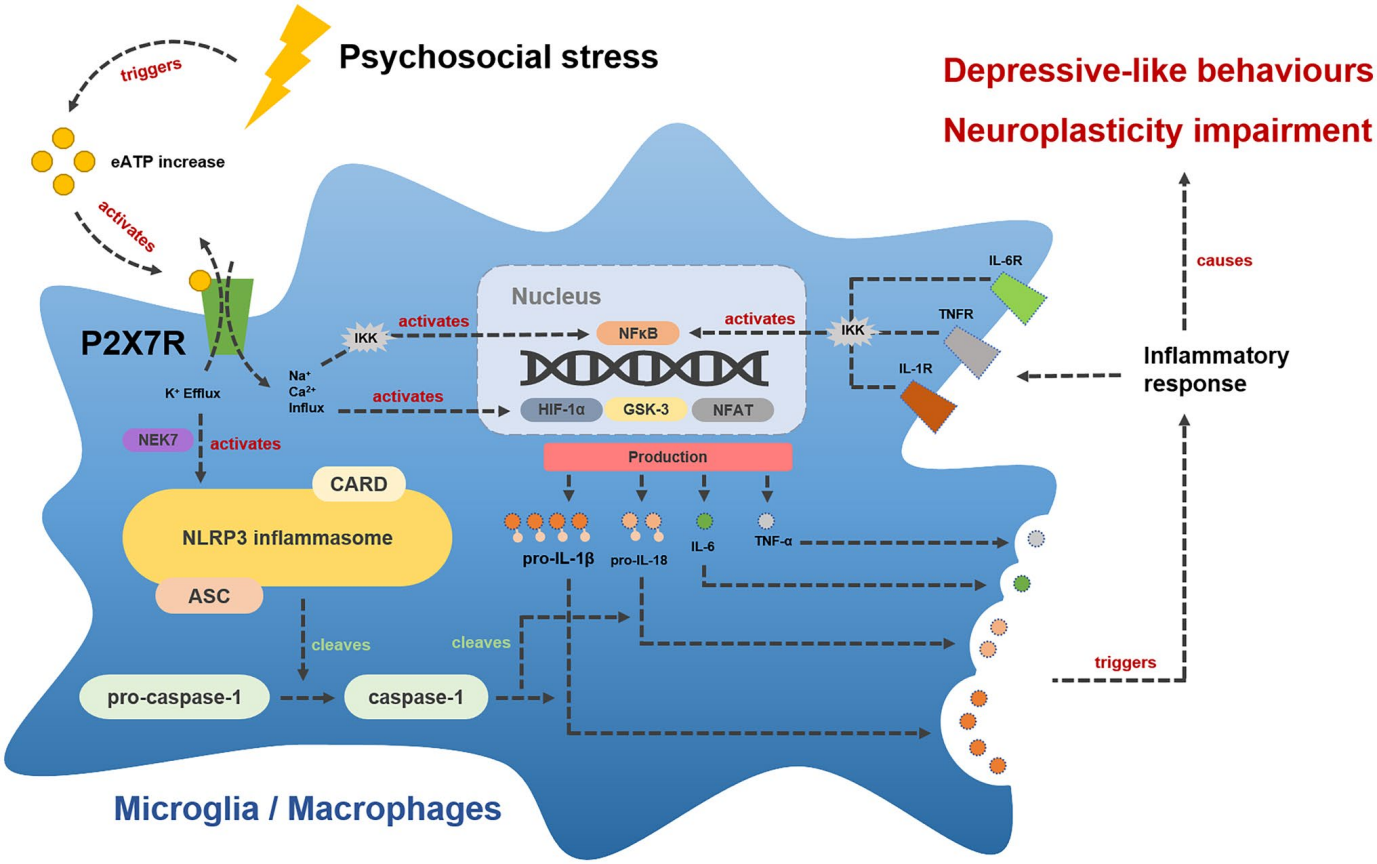
Hypothesized inflammatory pathways downstream of P2X7R in M/Ms following psychosocial stress. Upon psychosocial stress exposure, neurons and astrocytes release ATP into the extracellular space (eATP). This activates P2X7R on microglia and macrophages and causes NLRP3 assembly, caspase-1 activation and NFƙB upregulation. These mechanisms jointly cause increased IL-1β, IL-18, IL-6 and TNF-α production and release. Ultimately, this leads to a cellular and humoral inflammatory response, neuroplasticity impairment and depressive-like behaviour. *NEK7* NIMA-related kinase protein 7, *CARD* caspase activation and recruitment domain, *ASC* apoptosis-associated speck-like protein containing a CARD, *NFkB* nuclear factor ‘kappa-light-chain-enhancer’ of activated B-cells, *IKK* IκB-kinase-complex, *HIF-α* hypoxia-inducible factor 1-alpha, *GSK-3* glycogen synthase kinase 3, *NFAT* nuclear factor of activated T-cells, *IL* interleukin, *TNF-α* tumor necrosis factor alpha, *cytokine receptor* IL-R or TNFR

Intracellularly, signal transduction is facilitated by the P2X7R-induced potassium concentration decline. This induces the NIMA-related kinase 7 (NEK7) dependent assembly of the NLRP3 inflammasome [121], a multiprotein complex causing caspase-1 activation and the release of proinflammatory cytokines like IL-1β and IL-18 from astrocytes and microglia [38, 94, 109, 122, 123]. In addition, calcium influx upregulates the cellular energy metabolism by stimulating glycolysis as well as oxidative phosphorylation and causes further glutamate and ATP release from microglia and astrocytes, leading to heightened excitotoxicity [34, 36]. Aside from this, P2X7R activates several other inflammatory pathways including nuclear factor ‘kappa-light-chain-enhancer’ of activated B-cells (NFƙB), which jointly cause increased pro-IL-1β and pro-IL-18 transcription as well as the production of IL-6, IL-1α and TNF-α [109, 123]. The P2X7R mediated IL-1β release also induces an array of inflammatory effector molecules including cyclooxygenases, eicosanoids and reactive oxygen as well as nitrogen species [34, 109, 124, 125]. The pathways following P2X7R stimulation in M/Ms are illustrated in Fig. 1. Taken together, the multiple cellular and humoral immune processes downstream of P2X7R may translate CPSS/CPPS into the hallmark symptoms and neurobiological aberrations of depression [15–17, 33–35, 76, 116, 126–128].

## The P2X7R-NLRP3-IL-1β cascade mediates depressive-like behaviour

To support the importance of the P2X7R-NLRP3-IL-1β cascade in mediating depressive-like behaviour, we present the evidence for each single pathway component.

Chronic (21 days) bilateral, stereotactic injection of ATP or 2'(3')-*O*-(4-benzoylbenzoyl)-ATP (BzATP) into the hippocampus of male Sprague–Dawley rats led to depressive-like behaviour in the form of reduced rearing times and total distance travelled in the open field test (OFT) and decreased struggling and increased immobility in the tail suspension test (TST). Correspondingly, P2X7R knockout caused stress-resilience in the forced swim test (FST) and OFT compared to C57BL/6 mice when exposed to a 5-week CUS regime [113]. Basso et al*.* [129] also found antidepressant-like effects of P2X7R inactivation in the FST and TST. In a 12-week UCMS model in male Wistar rats, Pan et al. [130] observed a significant upregulation of NLRP3 and NFƙB signalling along with increased IL-1β serum und PFC levels. Wang et al*.* [88] performed a similar UCMS paradigm in male Sprague–Dawley rats and reported depressive-like behaviour in the form of decreased struggling in the FST, reduced rearing and locomotor activity in the OFT and decreased open arm time in the elevated plus maze (EPM). In a related approach, Zhang et al*.* [131] demonstrated that a pre-treatment of 8-week-old, male BALB/c mice with the NLRP3 inhibitor VX-765 inhibited the development of depressive-like behaviours. Compared to untreated but stressed controls, the VX-765 group showed no changes in the sucrose preference test (SPT) nor TST after 4 weeks of UCMS. Serum and hippocampal IL-1β levels as well as NLRP3 and caspase-1 measures remained unaltered in the VX-765 UCMS group. Similar effects were observed following 30 days of immobilisation stress in male NLRP3-null C57BL/6 mice. These animals did not show ATP or IL-1β level increase in the PFC or hippocampus and social interaction, food intake as well as performance in the SPT and FST remained unaltered [116]. Meanwhile, injection of recombinant IL-1β into the ventricle of Sprague–Dawley rats caused decreased sucrose intake in the SPT, increased immobility in the TST and supressed social interaction [132]. Vice versa, IL-1β antibody treatment during a 2-week CUS regime prevented depressive-like behaviour in the SPT and EPM compared to control mice receiving unspecific immunoglobulins (IgG) [94]. Analogously, 5 weeks of UCMS failed to induce behavioural or endocrine changes in male, IL-1 receptor knockout mice, while the same UCMS reduced neurogenesis, decreased sucrose preference in the SPT and lowered social exploration in C57BL/6 controls [133]. In line with these findings, intraventricular TNF-α injection induced depressive-like behaviour measured in the FST and TST in 6-week old, male C57BL/6 mice [134], while chronic administration of a TNF-α inhibitor (Infliximab 5 mg/kg/week) was able to attenuate depressive-like behaviour in the FST, EPM and SPT after 8 weeks of UCMS [56, 135]. TNF receptor 1 knockout mice also showed significantly decreased despair in the FST and TST compared to wild-type animals [134]. Last, application of an IL-6 antibody in male C57BL/6 mice exposed to 10 days of CSD reduced immobility in the TST and increased sucrose intake in the SPT [136]. Similarly, IL-6 knockout mice displayed reduced despair in the FST and TST, enhanced sucrose preference in the SPT and a partial resistance to learned helplessness compared to controls [137]. In summary, the evidence available demonstrates the importance of each component along the P2X7R-NLRP3-IL-1β cascade in mediating depressive-like behaviours and, when pharmacologically or genetically inactivated, in stress resilience.

## P2X7R antagonists in murine chronic stress models

We identified a total of four studies, namely Farooq et al. 2018, Aricioglu et al. 2009, Yue et al. 2017, and Iwata et al. 2016, which used P2X7R antagonists in CPSS/CPPS-based murine models of depression (Table 1).

**Table 1:** Compilation of methods and results of studies using P2X7R antagonistic drugs in murine depression models based on CPSS/CPPS application

Stress model and animal characteristics						P2X7R-antagonist and behavioural assessment			Main results	References
Stress paradigm/individual stressors		Stressor application	Paradigm duration	Species/strain	Sex/age	P2X7R antagonist and dosage/application and timing		Behavioural tests and timing/experimenter blinding		
Unpredictable Chronic Mild Stress (UCMS)	Social stress, wet bedding, frequent bedding change, cage tilting at 45°, place rat droppings in mice cages, playing predator sounds, altering day and night cycles, restraint stress (30 min)	Two stressors per day, over time increased to four or five stressors per day; random order	9 weeks	Mouse/BALB/cByJ	Male/7 weeks	BBG 50 mg/kg i.p	Application commenced 2 weeks after the UCMS start; performed until the end of UCMS	CSS (once weekly during the UCMS), NBS (assessed during the last week of UCMS; for more details see [138]) Experimenters were blinded to the treatment status for the CSS	UCMS resulted in (a) impaired CSS and NBS at weeks 8 and 9; improved by BBG (*p* < 0.01 vs. UCMS + saline group) (b) increased microglial activation and P2X7R expression in limbic and cortical areas; ameliorated by BBG (*p* < 0.05 vs. UCMS + saline group) (c) HPA dysfunction; attenuated by BBG (*p* < 0.05 vs. UCMS + saline group)	[139]
Unpredictable Chronic Mild Stress (UCMS)	Cage tilting (24 h), wet bedding (24 h), swimming in 4 °C cold or in 45 °C hot water (each 5 min), pairing with other stressed animal (48 h), level shaking (10 min), nip tail (1 min), inversion light/dark cycle (24 h)	One stressor per day; each stressor applied 5–6 times in total; the same stressor was not applied two days in a row; random order	6 weeks	Rat/Wistar-Albino	Male/8–10 weeks	BBG 25 mg/kg i.p BBG 50 mg/kg i.p	Application every 24 h during the last 3 weeks of UCMS	FST, SPT (both performed after UCMS ended) Experimenters were blinded to the treatment status for the FST	UCMS resulted in (a) anhedonic behaviour in SPT; attenuated by 25 and 50 mg BBG (*p* < 0.01 vs. UCMS + saline) (b) elevated immobility in the FST; recovered by 50 mg but not 25 mg BBG (*p* < 0.01 vs. UCMS + saline) (c) mRNA level increase of P2X7R, caspase-1, ASC, NFƙB, IL-1β, IL-6; attenuated by 50 mg BBG (*p* < 0.05 vs. UCMS + saline); microglia activation (Iba-1 in IHC; hippocampus) in the UCMS group; reduced by both 25 and 50 mg BBG (*p* < 0.01 vs. UCMS + saline)	[140]
Chronic Unpredictable Stressors (CUS)	Water/food deprivation (each 40 h), light–dark cycle reversal, hot environment (40 °C, 5 min), swimming in cold water (4 °C, 5 min), cage shake (30 min)	One stressor per day; random order	3 weeks	Rat/Sprague–Dawley	Male/n.a.*	BBG 1 μl (1 pM) A-438079 1 µl (1.75 nM) Bilateral microinjection into the hippocampus via an implanted cannula	Application once daily during CUS; injections were performed 30 min after each stress exposure	FST, OFT (both performed on the first day of CUS before the injection and on the last day of the CUS paradigm 30 min after the last injection occurred) Experimenters were blinded to the treatment status for all tests	CUS resulted in (a) elevated hippocampal extracellular ATP, increased cleaved-caspase-1, ASC and NLRP3 levels (*p* < 0.05 vs. controls); slightly raised IL-1β, unaltered P2X7R levels at weeks 1, 2 and 3 (*p* > 0.05 vs. controls) (b) depressive-like behaviour with reduced rearing and distance travelled in the OFT, less struggle and more immobility time in the FST (*p* < 0.05 vs. CUS + saline); CUS effects were attenuated by injection of BBG or A-438079 (*p* < 0.05 vs. CUS + saline)	[113]
Chronic Unpredictable Stressors (CUS)	Cage tilt, light–dark cycle change, crowd, odor, cold stress, no bedding, wet bedding, isolation, food/water deprivation, stroboscope, forced swim, cage rotation, immobilization stress	Two stressors per day; random order	8 weeks	Rat/Sprague–Dawley	Male/n.a	A-804598 5 mg/kg i.p	Application twice daily in the last 4 weeks of CUS (∑ 10 mg/kg/d of A-804598)	SPT, NSFT, EPM (all performed after UCMS ended) Experimenters were blinded to the treatment status for all tests	CUS resulted in (a) reduced consumption in the SPT, elevated latency to feed in the NSFT and reduced time in the open arms of the EPM (*p* < 0.05 vs. unstressed controls) (b) A-804598 significantly reversed the CUS effects in the SPT, NSFT and EPM (*p* < 0.05 vs. CUS group)	[94]

*BBG* brilliant blue G, *i.p.* intraperitoneal, *n.a.* not available, *ASC* apoptosis-associated speck-like protein containing a CARD

*CSS* coat state score, *NBS* nest building score, *SPT* sucrose preference test, *FST* forced swim test, *OFT* open field test, *NSFT* novelty suppressed feeding test, *EPM* elevated plus maze, *SCT* sucrose consumption test, *IHC* immunohistochemistry

*Rats were 180–200 g

To induce depressive-like behaviour, two of the studies applied a UCMS paradigm, while the others used CUS. We could not find a single study that employed a CSD paradigm. The stimuli used in the different stress paradigms are almost identical, but reporting detail varied between studies. This was particulary true regarding the stimulus duration and the total amount of time animals were exposed to either one or several stimuli. All studies reported random stressor application and employed at least one stressor per day. Exclusively Farooq et al. 2018 gradually increased stressor density over the course of their 9 weeks UCMS paradigm to five applications per day. In regards to the stressfull stimuli and their composition, the studies partially overlap. All disrupted the circadian rhythm and applied cage shake, tilt or rotation. Three studies used temperature-based stressors and half-employed immobilsation or food and water deprivation. Stressors like tail nip or stroboscope application were only found in one study. Psychophysical stressors were predominant in all stress paradigms, while psychosocial ones like predator sounds, social isolation or crowding were used in only half the studies to a minor and negligible degree. Stress paradigm duration was 3, 4, 8 or 9 weeks and thus differed between studies.

All studies exclusively included male animals. One study made use of BALB/cByJ mice, while the other three utilized either Sprague–Dawley or Wistar-Albino rats. Animal age at the start of UCMS or CUS varied between 7 and 10 weeks. However, Yue and Iwata et al*.* did not report the animals’ age. Animal numbers per experimental group ranged between 7 and 15.

In terms of P2X7R antagonist and dosage, Farooq and Aricioglu et al. injected Brilliant Blue G (BBG) intraperitoneal (i.p.) at 25–50 mg/kg, while Yue et al*.* microinjected either BBG or A-438079 into both hippocampi. Exclusively Iwata et al. 2016 administered a novel, selective P2X7R antagonist called A-804598 i.p. with 5 mg/kg. Antagonist application timing and frequency differed between the studies, with a tendency towards a single administration per day in the second half of the respective stress regime.

Experimenter blinding was reported in all studies, however, varying in extent. Behavioural assessment was non-uniform among the studies: an overlap was only found for the FST and the SPT, which were applied in half the studies. Beyond this, the following tests were each applied in one single study: OFT, EPM, Coat State Score, Nest Building Score, Novelty Suppressed Feeding Test (NSFT). Throughout the studies, behavioural testing was performed after or during the last week of UCMS or CUS. Only Yue and colleagues performed testing using the FST and OFT both on the first and last day of their 3-week CUS paradigm.

Following chronic stress application, the studies reported findings consistent with depressive-like behaviour. In addition, neurobiological aberrations in the form of cellular as well as humoral immune changes in the blood and in frontolimbic areas were found. It must be noted that Iwata et al*.*2016) did not reportthese changes following their CUS regime, but in a separate subsection of their study employing acute immobilisation stress. Half the studies showed a significant increase of P2X7R and NLRP3 expression alongside considerable M/Ms activation. Similarly, an upregulation of proinflammatory (NFƙB, IL-1β, IL-6) and apoptosis-related markers was found. Without exception, P2X7R antagonist application led to a significant attenuation of the UCMS- and CUS-induced neurobiological alterations and depressive-like behaviours. Aricioglu et al. (2009) even reported dose-dependent effects of BBG, with 50 mg/kg being more effective in reducing immobility in the FST than 25 mg/kg. None of the studies reported any side effects of BBG, A-438079 or A-804598 administration.

## Discussion and perspectives

The four studies jointly demonstrate that CUS and UCMS induces depressive-like behaviour in rodents. The observed behavioural aberrations were accompanied by frontal and limbic M/Ms activation and NLRP3-/NFƙB-mediated IL-1β increase. Moreover, the administration of a P2X7R antagonist ameliorated the stress effects on a cellular, humoral and behavioural level throughout the different experimental settings. These findings are in line with existing evidence demonstrating that CPSS/CPPS models like CUS, UCMS, CSD, chronic restraint or social isolation lead to NLRP3-/NFƙB-related cytokine release (IL-1β, IL-6, TNF-α) [35, 88, 130, 133, 141] and M/Ms activation, primarily in limbic structures like the PFC, hippocampus and nucleus accumbens [90, 91, 95, 130, 142–145].

In addition, and consistent with studies of NLRP3-, P2X7R- and IL-1β knockout strains [94, 113, 116, 129, 133, 146], chronic P2X7R antagonist injection led to considerable antidepressive and anti-inflammatory effects in each study. Similar effects were reported by Ribeiro et al*.* 2019, Catanzaro et al*.* 2014 and in a subsection of Iwata et al*.* 2016 in the context of acute stress [67, 94, 147, 148]. Related studies from other immunological research like alcohol-induced cirrhosis, rheumatoid arthritis and multiple sclerosis demonstrate comparably beneficial and anti-inflammatory properties of P2X7R antagonists [43, 107, 149, 150]. Overall, these results and commonalities with related studies substantiate the upcoming notion of inflammation as an important element in depression genesis [15, 16, 26, 27, 151].

Modelling depression in animals is traditionally based on the application of CPSS/CPPS or certain biological stressors during development, adolescence and adulthood [152]. In the light of three core validity criteria (construct/face/predictive), each different method can at least model one distinct environmental, neurobiological, or behavioural feature of depression [152, 153]. Although no single approach can resemble all disease factors, chronic, stress-based models like UCMS or CUS are considered the most valid options in reflecting disease complexity overall [13, 54–56]. However, the models reviewed here mostly lack psychosocial stressors and thus are much more CPPS than CPSS models [25, 154]. In this regard and in line with existing research [155–157], Du Preez et al. [158] previously showed distinct behavioural and neurobiological differences between CPSS, CPPS and combined CPSS/CPPS paradigms in male mice. Repeated saline injections led to anxiety-like behaviour in the OFT and NSFT, increased M/Ms activation and reduced TNF-α serum levels, corticosterone reactivity and hippocampal neuroplasticity. Meanwhile, social isolation resulted in depressive-like behaviour in the SPT as well as FST, increased hippocampal plasticity and elevated serum TNF-α, accompanied by decreased IL-1β, corticosterone reactivity and M/Ms density. Intriguingly, the combination of stressors led to yet another phenotype with increased anxiety-like features, reduced serum IL-1β levels and hippocampal plasticity but without significant alterations in corticosterone reactivity and M/Ms activation. These findings indicate that the biological and behavioural response strongly depends on the quality and composition of chronic stressors.

Despite the major role of CPSS in depression pathogenesis in humans, no translational studies have so far evaluated the effects of P2X7R antagonists in a CPSS-based depression-model like CSD. To measure stress effects, the reviewed studies used well-established behavioural tests. However, test battery composition was heterogenous and performed based on an endophenotype-driven hypothesis, increasing the risk of constraint and selection. In principle, neurocognitive and behavioural assessment needs to evolve past single test limitations and work towards a comprehensive approach to capture the entirety of psychosocial alterations of a distinct neuropsychiatric phenotype [13, 159]. Though different sentiments exist on how to best assess and class animal models and behaviours [159], the National Institute of Mental Health Research Domain Criteria (RDoC) are a fine-grained matrix interconnecting complex neurobiological aberrations and behavioural domains [55, 153, 160]. This enables precise, endophenotype-driven modelling of physiological systems involved in the pathogenesis of neuropsychiatric disorders. In addition, due to the interspecies homology within the RDoC domains, findings can be translated from animals to humans and vice versa [13, 14].

On a related note, the studies at hand only used male animals at the verge of adulthood. In neuropsychiatric research, considerable sex effects have been shown for animal behaviour varying by disease state, species and strain [161–163] as well as for the cellular response in general and M/Ms response in particular [163–166]. Moreover, contemporary studies have emphasized the need of a proper age-translation matrix between rodents and human in research [167, 168]. Future studies using P2X7R antagonists in murine depression models should include both sexes, account for age effects and employ standardized and comprehensive behavioural assessments. In addition to that, the widespread use of imprecise language in reporting behavioural results of neuropsychiatric animal models needs to be improved to reduce the risk of false interpretation primarily in the form of overgeneralisation [13, 153, 169, 170].

Our analysis of the pharmacological properties of the P2X7R antagonists used in the four studies is as follows: BBG, the most used substance, is a non-selective P2X7R antagonist, that penetrates the blood–brain barrier and, aside P2X7R, binds P2X1/2/3/4/5R and voltage-gated sodium channels [43, 108, 171, 172]. In general, antagonist affinity varies depending on the drug itself, the P2X-subtype, and the species as well as strain [173, 174]. The half maximal inhibitory concentration of BBG for P2X7R in rat and human cells lies between 10 and 200 nM, meaning it is up to 50-fold lower than for the other receptors [43, 107, 174]. It even acts 1000-times stronger on P2X7R than on P2X4R [174], making it a semi- rather than a non-selective antagonist. BBG has been used in a variety of research settings revolving around the role of P2X7R and M/Ms in neuroinflammation, which overall yielded positive and anti-inflammatory results [43, 175, 176]. However, a lack of in vivo pharmacodynamic analyses in regards to BBG-mediated P2X7R antagonism in the brain has been demonstrated, questioning the overall validity and interpretability of published data [176]. A-804598 and A-438079 can cross the blood–brain barrier and are selective P2X7R antagonists [43, 173, 177]. A-438079 has been used in several inflammatory disease models, causing beneficial effects [178]. However, due to its short biological half-life and limited bioavailability it is considered unsuitable for chronic or clinical application [108, 177, 179]. In contrast, A-804598 has been used in different inflammation models [30, 43, 149, 150, 180] as well as in acute stress [147, 148] and mania models [181]. Based on the limited evidence in the context of neuropsychiatry, the used antagonists can be considered contemporary and appropriate. However, the application of antagonists with different pharmacological properties in various stress paradigms allows only mixed receptor blockage along with paradigm-selective behavioural effects to be deducted. Results are, therefore, only partially comparable and must be interpreted with caution [108]. In the future, the attribution of study findings for P2X7R should be reserved for models using highly selective antagonists. Furthermore, studies should take the pharmacological properties, such as P2X7R affinity, into account and favour selective over non-selective drugs [176].

Altogether, we propose a strict commitment to the RDoC and to the newly introduced STRANGE framework (social background, trappability and self-selection, rearing history, acclimation and habituation, natural changes in responsiveness, genetic make-up, experience) in study planning and reporting in translational neuropsychiatry to minimize experimental bias, maximize animal usage, increase animal data quality and impact by enhancing reproducibility and generalizability overall [55, 153, 160, 182, 183].

## Conclusion

The four reviewed studies successfully induced depressive-like behaviour, immune changes (NLRP3 assembly, IL-1β level increase, M/Ms activation) and hippocampal neuroplasticity impairment by use of different chronic, psychophysical stress paradigms. P2X7R antagonist application (BBG, A-438079, A-804598) led to an attenuation of the stress-induced neurobiological and behavioural aberrations in all four studies. These findings highlight the potential of P2X7R modulation in chronic stress and depression. However, to advance our understanding of specific P2X7R-related effects, methodological refinements are needed. We propose a commitment to the RDoC and the STRANGE framework in study planning and reporting to reduce methodological and pharmacological heterogeneity, minimize bias and increase data validity, reliability, and generalizability. Ultimately, we believe this to be the roadmap to reform animal data impact and improve the life of depressed patients worldwide.

## Data Availability

To identify suitable research concerning the effects of P2X7 receptor antagonists on chronic stress effects in animal models, IVMH searched the electronic database PubMed using three broad keyword combinations: *(P2X7R antagonist) AND (chronic stress)*—> 13 results; *(P2X7R antagonist) AND (stress)*—> 41 results; *(P2X7 antagonist) AND (stress)*—> 97 results. Out of these available studies, only 4 met the inclusion criteria (i.e., chronic psychosocial or psychophysical stress model of depressive-like behaviour in animals; iterative P2X7 antagonist application). These publications are reviewed in this article.
